# Microsaccade biases can reflect task-specific spatial memorization strategies

**DOI:** 10.3389/fnins.2025.1526213

**Published:** 2025-03-31

**Authors:** Samson Chota, Kabir Arora, J. Leon Kenemans, Surya Gayet, Stefan Van der Stigchel

**Affiliations:** Experimental Psychology, Helmholtz Institute, Utrecht University, Utrecht, Netherlands

**Keywords:** internal attention, spatial attention, visual working memory (VWM), microsaccade direction, gaze bias

Previous work has suggested that small directional eye movements not only reveal the focus of external spatial attention toward visible stimuli but also accompany shifts of internal attention to stimuli in visual working memory (VWM, Van Ede et al., 2019). When the orientations of two bars are memorized and a subsequent retro-cue indicates which orientation needs to be reported, participants' gaze is systematically biased toward the former location of the cued item. This finding was interpreted as evidence that the oculomotor system indexes internal attention; attention directed toward the location where stimuli maintained in VWM were previously presented. Because the location of the bars is presumably not relevant to the memory report, this led the authors to conclude that different features in VWM (such as orientation) are automatically associated with different spatial locations, implying that VWM is inherently spatially organized. This conclusion, however, depends on the key assumptions that participants (1) indeed memorize and (2) subsequently attend orientation features. Here we re-analyse Experiment 1 by Van Ede et al. (2019) and demonstrate that this assumption may not hold. The gaze bias instead reveals that participants might deploy an alternative spatial-based strategy, memorizing the endpoint locations of the bars, thereby allowing them to solve the task without memorizing orientations. Although we do not call into question the conclusion that internal attention is inherently spatially organized, our results do imply that directional microsaccade biases might also reflect attention directed at task-relevant, location-specific stimulus properties, rather than reflecting internal attention directed at memorized orientations.

**Figure 1:**
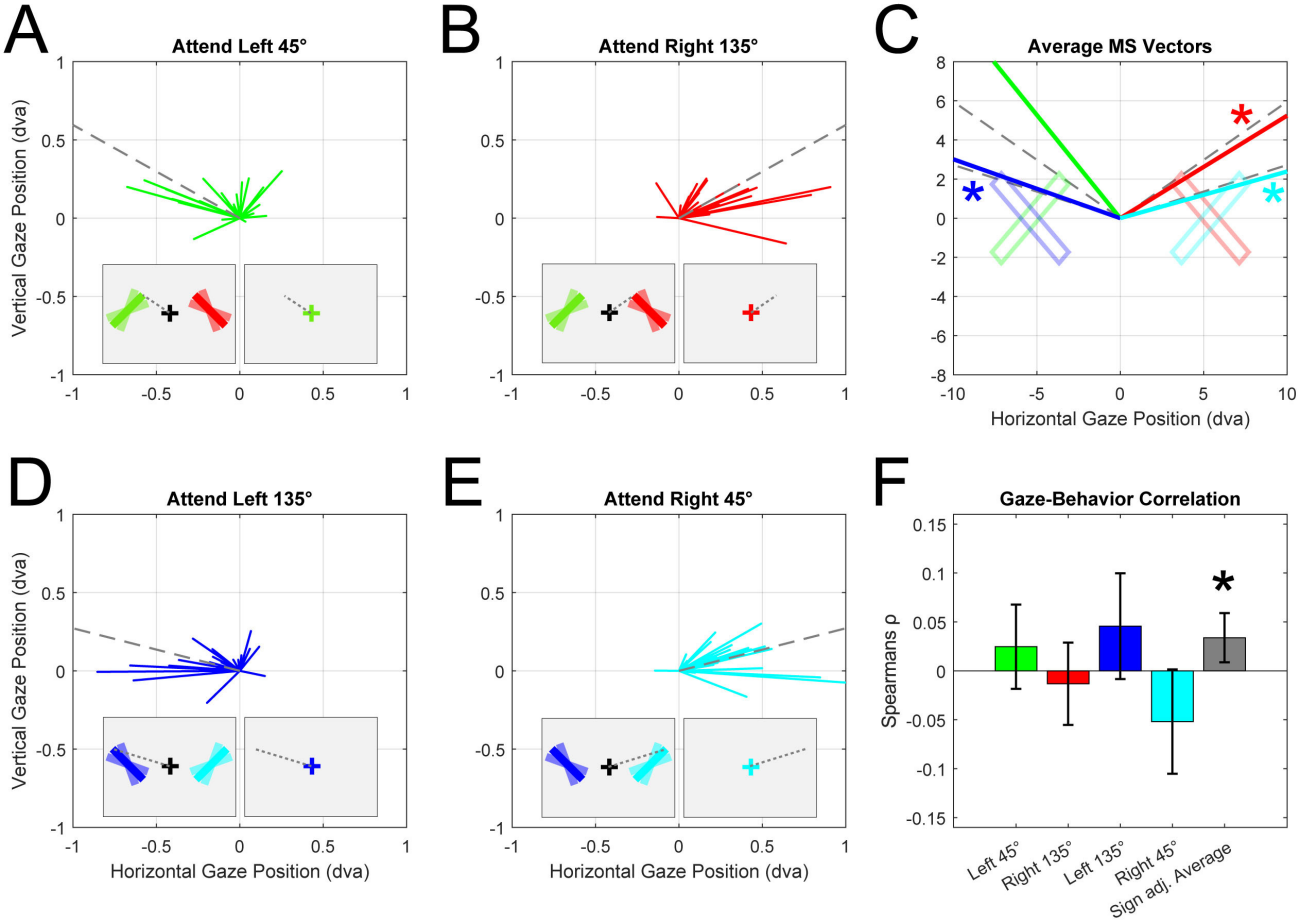
Microsaccade analysis from Experiment 1 by Van Ede et al. (2019; N = 23, trials included = 20,864). **(A)** Individual participants average microsaccade vectors (green lines) when left bar (orientation range: 20° to 70°, mean 45° relative to horizontal) was cued. Colored bars depict the maximal range of orientations. Dotted lines represent direction of upper endpoint. Microsaccades were detected between 400 ms and 1,000 ms after cue and were only included if their amplitude was smaller than 2 dva. Insets show simplified task sequence and orientation configuration of bar stimuli. **(B)** Average microsaccade vectors when right bar (orientation range: 110° to 160°, mean 135°) was cued. **(C)** Group average microsaccade vectors for all conditions (color-matched, see **A, B, D, E)**. Asterisks indicate conditions where microsaccade and bar endpoint direction did not differ significantly. **(D)** Average microsaccade vectors when left bar (orientation range: 110° to 160°, mean 135°) was cued. **(E)** Average microsaccade vectors when right bar (orientation range: 20° to 70°, mean 45°) was cued. **(F)** Individuals correlation coefficients per condition. Coefficients were calculated by correlating location memory error (angle difference between microsaccade direction and actual endpoint) with the report error (difference between reported orientation and true orientation). Gray bar reflects sign adjusted average correlation coefficients. Error bars indicate 95% confidence intervals.

Studies on internal attention using gaze have primarily used rectangular oriented bars (Van Ede et al., 2019; van Ede et al., 2020; Liu et al., 2022; Draschkow et al., 2022). Bar stimuli are usually rotated around their center of mass, which means that any orientation maps onto unique endpoints. Therefore, participants could memorize these endpoints instead of the orientation information *per se*. This strategy could serve as a form of cognitive offloading; akin to keeping a finger pointed at the former stimulus location, maintaining a microsaccade bias during the delay allows to “remember” a location with minimal mental effort. We tested if participants' microsaccade directions depended on the endpoint/orientation of the stimuli by grouping trials based on the location of the cued item (left vs. right) and the average orientation of the bars [45° (orientation range: 20° to 70°) vs. 135° (110° to 160°), relative to horizontal midline]. By doing so, we divided the original dataset into four new conditions of interest. In each condition, the endpoints of the bars occupied unique and distinguishable positions on the screen (see Figures 1A–E). For each of these conditions we detected and calculated the average direction of microsaccades occurring 400 to 100 ms after the cue. Visual inspection suggested that microsaccade direction coincided with the upper endpoint of the attended bar in all conditions. Statistical tests confirmed that microsaccades were biased away from the left and right bar centers in all conditions [circular *t*-test: Left 45° = 3.14^*^, 95% CI (1.39, 2.45), Right 135° = 0^*^, 95% CI (0.39, 1.01), Left 135° = 3.14^*^, 95% CI (2.11, 2.93), Right 45° = 0^*^, 95% CI (0.074, 0.63), units in radians, Figure 1C]. Moreover, microsaccade direction did not significantly differ from the direction of the bar endpoints located in the upper visual field in 3 out of 4 conditions [circular *t*-test: Left 45° = 1.92^*^, 95% CI (1.39, 2.45), Right 135° = 0.71, 95% CI (0.39, 1.01), Left 135° = 2.52, 95% CI (2.11, 2.93), Right 45° = 0.35, 95% CI (0.074, 0.63), Figure 1C]. Taken together, these results show that attention, as indexed through microsaccade biases, was specifically directed to the former location of the attended bar's “upper” endpoint, rather than its center of mass.

If participants indeed rely on memorized locations (i.e., bar endpoints) for their final report, errors in their memory should translate into corresponding errors in their gaze. For example, if participants memorized the upper endpoint of a bar oriented 45° on the right (Figure 1E, cyan bar), we expect their microsaccades to point toward the bar's endpoint at 15° (Figure 1E, dotted line). If microsaccades instead point toward a more clockwise direction (e.g., 10°), this could indicate that they erroneously memorized a different location, consistent with a more clockwise-oriented bar (oriented 30°). If participants indeed report orientation based on a memorized location—rather than orientation, we would expect their report to be biased clockwise relative to the true location (by 15°). This hypothesis predicts specific correlations for each condition due to the vertical symmetry of the stimulus display. To the left of fixation, larger clockwise microsaccade errors (saccade angle—endpoint angle) should result in larger clockwise report errors (reported angle—true angle) and thus a positive correlation. Conversely, to the right of fixation, larger clockwise microsaccade errors should lead to larger counterclockwise report errors and therefore a negative correlation. We tested this by performing within-subject trial-by-trial Pearson correlations between the report error and the microsaccade error. Group-level *t*-tests on the correlation coefficients indicated positive relationships in the Left 45° (rho = 0.025) and Left 135° (rho = 0.046) conditions and negative relationships in the Right 45° (rho = −0.052) and Right 135° (rho = −0.013, Figure 1F). When averaging these biases across all four conditions (sign-flipping the “Right” conditions), we found significantly positive correlations between gaze bias and report error across subjects. This highly specific pattern of results directly supports the hypothesis that participants infer the stimulus orientation from memorized locations. While we cannot completely exclude the possibility that participants also encoded orientation, the bias toward upper endpoints and the correlation between gaze and report errors, demonstrate that the spatial strategy was likely a significant driver of the observed gaze patterns.

Our results have an important implication for the interpretation of microsaccade biases, namely that these biases can reflect a stimulus specific spatial strategy that does not involve memorization of orientation *per se*. Despite these new implications, we do not call into question the main theoretical conclusions of Van Ede et al. (2019) that internal attention is inherently spatially organized. Since then, similar microsaccade biases were observed using stimuli without a clear endpoint (Ester and Weese, 2023; Liu et al., 2024; Arora et al., in press). In fact, we recently conducted a retro-cue paradigm like that of Van Ede et al. (2019) but using oriented Gabors instead of bars (Arora et al., in press) and found no evidence for an endpoint bias. Moreover, in their Experiment 4, Van Ede et al. (2019) showed that the horizontal microsaccade bias also emerges when participants need to report the color of a bar that was cued via its orientation (0° vs. 90°). In this case, maintaining attention (or gaze) at the bar endpoint is not a useful strategy for the task of reporting the color. In sum, there is ample support for the original conclusions of Van Ede et al. (2019) that visual working memory is inherently spatially organized.

To conclude, we call for caution in interpreting microsaccade biases, similar to those reported in the article of Van Ede et al. (2019), as unequivocally reflecting shifts of internal attention towards memorized orientation features. Particular stimuli and task designs may allow for alternative (e.g., spatial) memorization strategies and can evoke stimulus-specific gaze patterns (Hafed and Ignashchenkova, 2013; Willett and Mayo, 2023). Furthermore, and theoretically of greater interest, our results show that observers can approach even the simplest working memory recall tasks in a variety of ways. When set-up and stimuli allow for, observers may deploy unexpected but resource-efficient strategies, highlighting the richness of visuospatial working memory use.
